# Metal Dependence of Signal Transmission through Molecular Quantum-Dot Cellular Automata (QCA): A Theoretical Study on Fe, Ru, and Os Mixed-Valence Complexes

**DOI:** 10.3390/ma3084277

**Published:** 2010-08-06

**Authors:** Ken Tokunaga

**Affiliations:** General Education Department, Faculty of Engineering, Kogakuin University, Nakano-machi 2665-1, Hachioji, Tokyo 192-0015, Japan; E-Mail: tokunaga@cc.kogakuin.ac.jp

**Keywords:** quantum dot, automaton, QCA, mixed-valence complexes, Creutz-Taube complexes, quantum dynamics, Fe, Ru, Os, density functional theory

## Abstract

Dynamic behavior of signal transmission through metal complexes [L${}_{5}$M-BL-ML${}_{5}$]${}^{5+}$ (M=Fe, Ru, Os, BL=pyrazine (**py**), 4,4’-bipyridine (**bpy**), L=NH${}_{3}$), which are simplified models of the molecular quantum-dot cellular automata (molecular QCA), is discussed from the viewpoint of one-electron theory, density functional theory. It is found that for **py** complexes, the signal transmission time ($t_{\mathrm{st}}$) is Fe(0.6 fs) < Os(0.7 fs) < Ru(1.1 fs) and the signal amplitude (*A*) is Fe(0.05 e) < Os(0.06 e) < Ru(0.10 e). For **bpy** complexes, $t_{\mathrm{st}}$ and *A* are Fe(1.4 fs) < Os(1.7 fs) < Ru(2.5 fs) and Os(0.11 e) < Ru(0.12 e) < Fe(0.13 e), respectively. **Bpy** complexes generally have stronger signal amplitude, but waste longer time for signal transmission than **py** complexes. Among all complexes, Fe complex with **bpy** BL shows the best result. These results are discussed from overlap integral and energy gap of molecular orbitals.

## 1. Introduction

**Figure 1:**
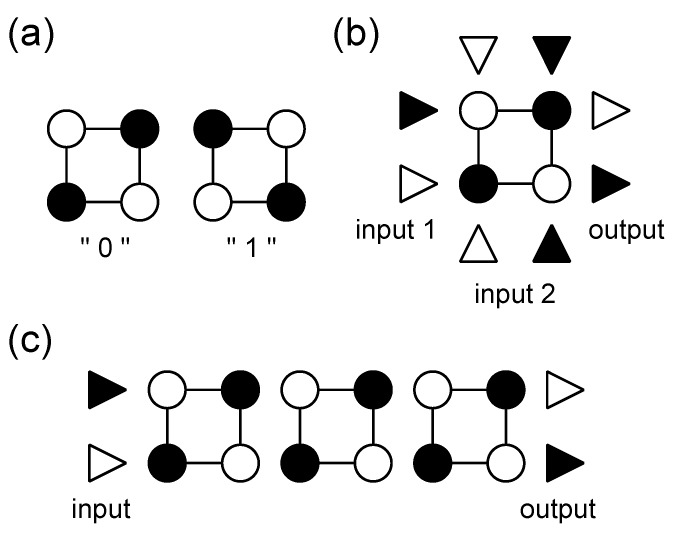
(a) Two degenerate states of QCA cell, "0" and "1". Some applications of QCA cell: (b) QCA logic gate (AND gate) and (c) QCA signal transmission wire. Charge of open circles and triangles is more positive relative to that of filled circles and triangles.

Quantum-dot cellular automata (QCA) device [1], which utilizes two degenerate states of metal dots “0” and “1” (Figure 1(a)) for operation, is one of next-generation devices which have been actively studied [2]. The QCA devices such as an AND logic gate (Figure 1(b)) and a signal transmission wire (Figure 1(c)) are expected to achieve a dramatic saving of energy and an increase in processing speed of computing since these devices are free from a current flow.

The success of several QCA device operations has been already reported [3,4]. For improvement in operation temperature and size of the devices, however, the idea of molecular quantum-dot cellular automata (molecular QCA) devices [5], in which a QCA cell constructed from small metallic dots is replaced by a single molecule, was proposed. Syntheses of tetranuclear complexes [6,7,8,9,10] and simplified dinuclear complexes [11,12], and single-molecule observation of the dinuclear complexes [13,14] have been investigated for the realization of molecular QCA devices. Also, theoretical simulations of QCA devices have been reported by many research groups [15,16,17,18,19,20,21]. However, the capacity of molecular QCA devices for molecular computing is still open.

**Figure 2:**
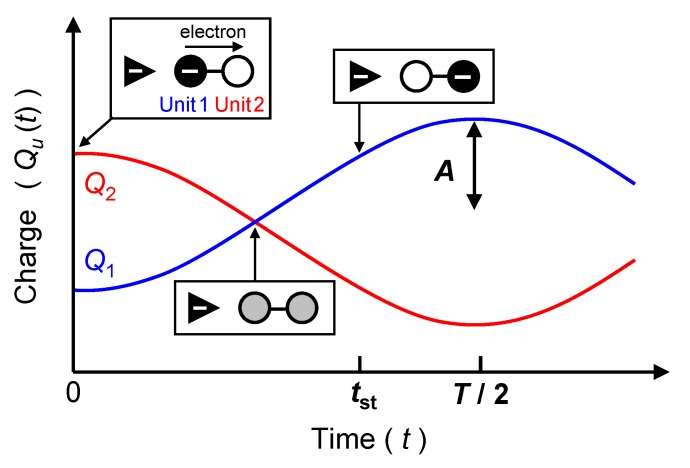
Simplified two site model of QCA cell and schematic picture of signal transmission between two units, unit 1 (U1) and unit 2 (U2). *A*, *T*, and $t_{\mathrm{st}}$ are the signal amplitude, the signal period, and the signal transmission time, respectively.

Very recently, I have proposed the simple method for an analysis of dynamic behavior of QCA devices, taking Creutz-Taube complexes [L${}_{5}$Ru-BL-RuL${}_{5}$]${}^{5+}$ (BL=pyrazine, 4,4’-bipyridine, L=NH${}_{3}$) as examples [22]. Using this method, main properties concerning the signal transmission such as the signal period *T*, the signal amplitude *A*, and the signal transmission time $t_{\mathrm{st}}$ (Figure 2) can be interpreted as follows: signal period (*T*) is inverse proportional to an energy gap between HOMO (the highest occupied molecular orbital, *H*) and LUMO (the lowest unoccupied molecular orbital, *L*) of the final stationary state, $\Delta \varepsilon_{HL}$. Signal amplitude (*A*) is proportional to an overlap integral between HOMO of the initial stationary state ($H^{\prime }$) and LUMO of the final stationary state (*L*), $d_{LH^{\prime }}$. Signal transmission time ($t_{\mathrm{st}}$) is determined depending on the balance of *A* and *T*. This method has advantage that signal transmission behavior can be analyzed from the viewpoint of one electron properties, which are shapes of molecular orbitals (MOs) and MO energies. Thus, the proposed method is suitable for simple design of high-performance molecular QCA.

In the present work, the proposed method [22] is applied to the simulation and analysis of metal dependence of signal transmission behavior through molecular QCA, taking [L${}_{5}$M-BL-ML${}_{5}$]${}^{5+}$ (M=Fe, Ru, Os, BL=pyrazine, 4,4’-bipyridine, L=NH${}_{3}$) as simplified models of the molecular QCA. Metal dependence of signal transmission is then discussed from the viewpoint of MO and the validity of the proposed method is also confirmed.

This paper is organized as follows. In section 2, computational model and method are shortly presented. The method for time evolution of the Mulliken charge [23] is briefly explained. In section 3, dynamic responses of molecular QCA cell upon the switch (*q* = +0.5e → *q* = -0.5e), that corresponds to one-electron injection to the input, are calculated based on the density functional theory (DFT). In section 4, dynamic properties of molecular QCA cell are discussed from the viewpoint of MOs and orbital energies. Finally, this work is summarized in section 5.

## 2. Computational

### 2.1. Model

**Figure 3:**
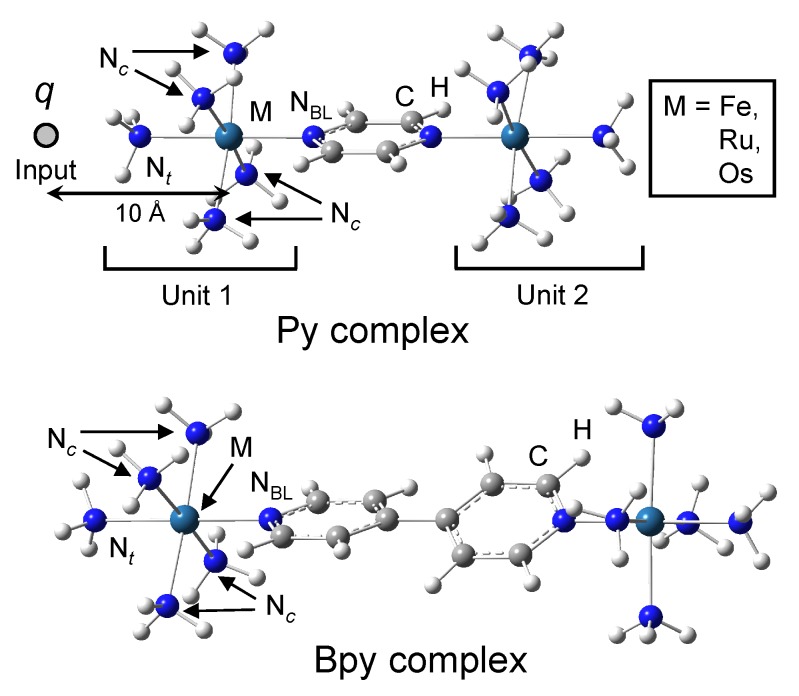
Schematic structures of **py** and **bpy** complexes. Input *q* is placed at a distance $r_{q-M}$ = 10 Å.

Dinuclear complexes, [L${}_{5}$M-(BL)-ML${}_{5}$]${}^{5+}$, shown in Figure 3 are selected to understand the metal dependence of signal transmission through the molecular QCA cell. Metals (M) of the complexes are selected as Fe, Ru, and Os. Bridging ligand (BL) of the complexes is pyrazine (**py**) or 4,4’-bipyridine (**bpy**), and ligand (L) is NH${}_{3}$. Total charge of the whole molecule is +5, excluding the input point charge *q*. These molecules are well-known as mixed-valence complexes such as Creutz-Taube complexes [24,25]. Point charge *q* placed parallel to M-N${}_{\mathrm{BL}}$ axis at a distance of $r_{q-M}$ = 10 Å from the M atom is used as an input to the complexes. Upon the switch of input, point charge is suddenly changed from +0.5e to -0.5e. Unit 1 (U1) is constructed from one M atom near to the input plus five NH${}_{3}$ ligands, and unit 2 (U2) is constructed from one M atom far from the input plus five NH${}_{3}$ ligands.

### 2.2. Method

The method for time evolution of unit charge has been already shown in my previous paper [22], so that the method is briefly explained here. In initial and final stationary states, the following one-electron equations

(1) $$\begin{array}{c} h^{i}|\psi_{n}^{i}\rangle =\varepsilon_{n}^{i}|\psi_{n}^{i}\rangle ,\;h^{f}|\psi_{n}^{f}\rangle =\varepsilon_{n}^{f}|\psi_{n}^{f}\rangle \end{array}$$

are satisfied, where *h*, $|\psi_{n}\rangle$, and $\varepsilon_{n}$ denote one-electron Hamiltonian, *n*th MO, and *n*th orbital energy, respectively. Superscripts “i” and “f” mean initial stationary state when *q* = +0.5e and final stationary state when *q* = -0.5e, respectively.

Expanding the initial state $|\psi_{n}^{i}\rangle \;(=|\psi_{n}\left(t=0\right)\rangle )$ by the complete set of $|\psi_{n}^{f}\rangle$ and adopting an approximation [22], one electron wave function at a time *t* is written as

(2) $$\begin{array}{c} |\psi_{n}\left(t\right)\rangle =\sum_{j}^{\mathrm{all}}|\psi_{j}^{f}\rangle e^{-i\;\varepsilon_{j}^{f}\;t}d_{jn}, \end{array}$$

where $d_{jn}=\langle \psi_{j}^{f}|\psi_{n}\left(0\right)\rangle =\langle \psi_{j}^{f}|\psi_{n}^{i}\rangle$. Total number of electrons, *N*, is represented as

(3) $$\begin{array}{ccc} N & = & \sum_{n}^{\mathrm{occ}.}\langle \psi_{n}\left(t\right)|\psi_{n}\left(t\right)\rangle =\sum_{\mu ,\nu }^{\mathrm{all}}P_{\nu \mu }S_{\mu \nu }, \end{array}$$

(4) $$\begin{array}{ccc} P_{\nu \mu } & = & \sum_{n}^{\mathrm{occ}.}\sum_{j,j^{\prime }}^{\mathrm{all}}d_{jn}d_{j^{\prime }n}\cdot c_{j\mu }c_{j^{\prime }\nu }\cdot \cos\left(\Delta \varepsilon_{jj^{\prime }}\;t\right), \end{array}$$

(5) $$\begin{array}{ccc} S_{\mu \nu } & = & \langle \phi_{\mu }|\phi_{\nu }\rangle , \end{array}$$

where *S*, *P*, $\phi_{\mu }$, $c_{j\mu }$, $\Delta \varepsilon_{jj^{\prime }}$, and *t* mean overlap matrix, population matrix, *μ*th atomic orbital (AO), coefficients of *μ*th AO of *j*th MO, energy gap between *j*th and $j^{\prime }$th MOs, and time after the moment of the switch, respectively. *N* is constant for the whole molecule, but is time-dependent for each unit. Time-dependent Mulliken charge of unit *u* is defined as

(6) $$\begin{array}{c} Q_{u}\left(t\right)=\sum_{a\in u}^{\mathrm{Atom}}\left\{Z_{a}-\sum_{\nu \in a}^{\mathrm{Basis}}{\left(\mathrm{PS}\right)}_{\nu \nu }\right\}, \end{array}$$

where $Z_{a}$ is a nuclear charge of an atom *a*. The first summation is taken over all atoms included in unit *u*. The value in the braces of Equation 6 corresponds to the Mulliken charge of an atom *a*.

All dynamic calculations were performed by the unrestricted DFT method using B3LYP functional. Hartree-Fock (HF) calculations were also checked, but detailed results are not shown in the text. Conventional basis set was used for H, C, and N atoms (6-31G(d) for C and N atoms, and 6-31G for H atoms). All-electron 3-21G basis set was used for Fe and Ru atoms, and LANL2DZ basis set and LANL2 pseudo potential were used for Ru and Os atoms. It was confirmed about Ru complexes that there is only a small difference between the results obtained by 3-21G and LANL2DZ basis sets. Therefore, the comparison between Fe(3-21G), Ru(3-21G), and Os(LANL2DZ) complexes will be valid. Geometrical optimizations and self-consistent field electronic calculations were performed by the Gaussian 03 program package [26].

## 3. Results

### 3.1. Geometric Structures

Calculated geometric parameters of **py** and **bpy** complexes are shown in Table 1, respectively. N${}_{\mathrm{BL}}$, N${}_{c}$, and N${}_{t}$ represent N atoms of M-BL, cis-M-NH${}_{3}$, and trans-M-NH${}_{3}$ bonds, respectively. In this work, all possible symmetries (including $C_{1}$ point group) were checked in the research of the stable structures, and it was confirmed that the most stable structures have no vibrational modes with imaginary frequencies.

**Table 1:** Summary of symmetries, irreducible representations of electronic state, and computed M-N bond lengths (Å) of **py** and **bpy** complexes. M-N${}_{c}$ bond length is averaged over all M-N${}_{c}$ bonds.

	py				bpy		
	Fe	Ru	Os		Fe	Ru	Os
Symmetry	$C_{2}$	$C_{2h}$	$C_{2}$		$C_{2}$	$C_{2}$	$C_{2}$
Electronic State	${}^{2}B$	${}^{2}B_{g}$	${}^{2}B$		${}^{2}B$	${}^{2}B$	${}^{2}B$
M-N${}_{\mathrm{BL}}$	1.939	2.206	2.099		1.927	2.169	2.115
M-N${}_{c}$	2.028	2.210	2.197		2.026	2.205	2.192
M-N${}_{t}$	2.075	2.191	2.211		2.071	2.208	2.214
dihedral angle	-	-	-		15.1	28.3	23.0

For **py** complexes, imposing $C_{2h}$, $C_{2v}$, $C_{2}$, $C_{s}$, and $C_{i}$ symmetries, the most stable symmetries were obtained as $C_{2h}$ symmetry (${}^{2}B_{g}$ state) for Ru complex and $C_{2}$ symmetry (${}^{2}B$ state) for Fe and Os complexes. Therefore, in one complex, two M atoms of the complex are equivalent so that **py** complexes are regarded as Class III of Robin-Day’s classification [27].

For all **bpy** complexes, the most stable symmetries were obtained as $C_{2}$ symmetry (${}^{2}B$ state). The dihedral angles between two C${}_{5}$N rings are 15.1${}^{\circ }$, 28.3${}^{\circ }$, and 23.0${}^{\circ }$ for Fe, Ru, and Os complexes, respectively. DFT calculation predicts **bpy** complex also to be classified into Class III.

It should be noted that Ru complex with **bpy** ligand is classified into Class II by the experiment [28]. In my previous paper [22], **bpy** complexes were classified into Class III and Class II by DFT and HF methods, respectively. And it was found that signal transmission does not take place in Class II complex by HF method. Therefore, I focused only on the Class III result by DFT method in order to analysis signal transmission behavior and expand knowledge about molecular design of QCA even though the classification of **bpy** complex into Class III is contradict to the experimental observation. The same tendency was obtained for Fe and Os complexes in the present work. Signal transmission does not take place in Class II **bpy** complex by HF method (not shown in the text). Therefore, I again focus my attention on the analysis of Class III **bpy** complex by DFT method in order to check the validity of analysis method proposed in my previous paper [22] and to expand knowledge about QCA.

### 3.2. Electronic Structures

**Figure 4:**
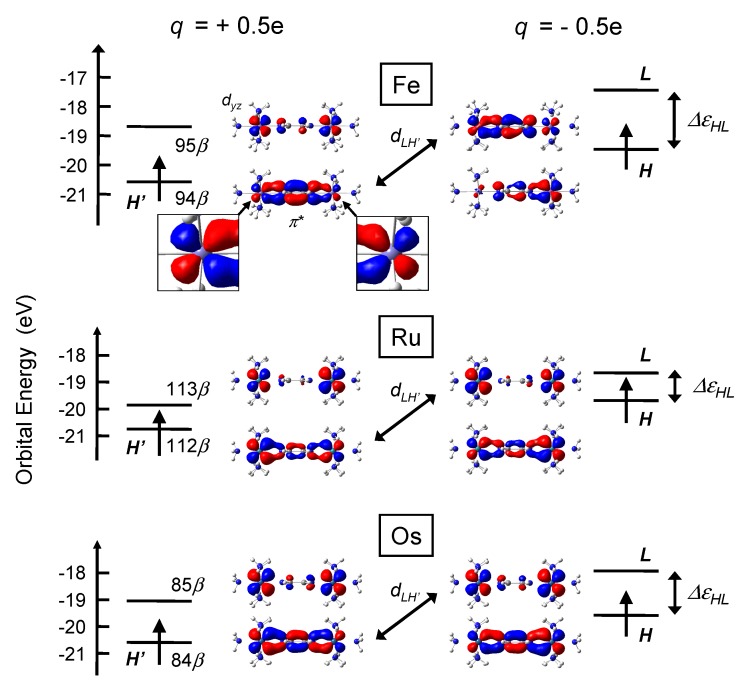
HOMO and LUMO with *β* spin of **py** complex when q=+0.5e (left) and q=-0.5e (right).

**Figure 5:**
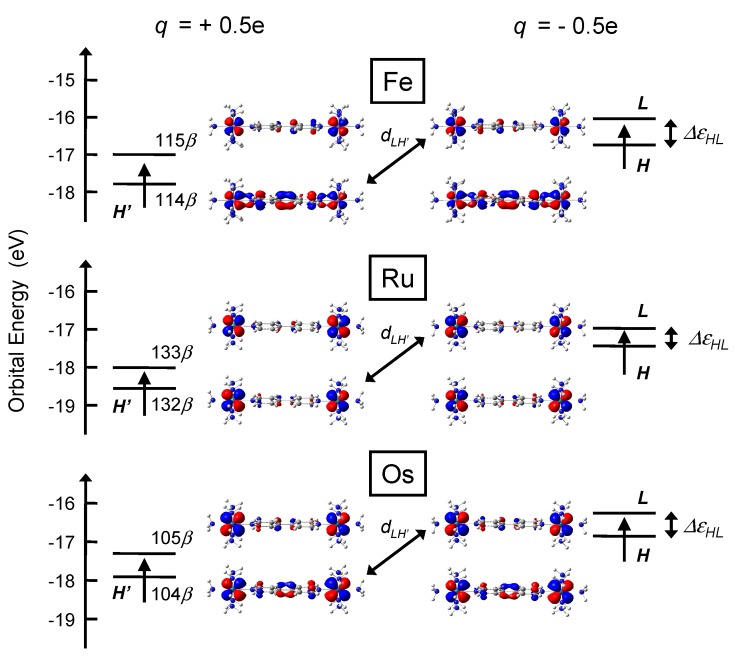
HOMO and LUMO with *β* spin of **bpy** complex when q=+0.5e (left) and q=-0.5e (right).

Change in the input charge from *q* = +0.5e to *q* = -0.5e, which corresponds to one-electron injection to the input, is considered. Figure 4 and Figure 5 show frontier MOs and orbital energies of stationary states of **py** and **bpy** complexes before (left) and after (right) the switch of the input. Only HOMO and LUMO with *β* spin are shown here since other orbitals plays almost no role in signal transmission [22]. These MOs are mainly constructed from $\pi^{*}$ orbital of BL and $d_{yz}$ orbital of M atom. HOMOs have larger distribution on U1 when q=+0.5e due to the coulombic attraction (See the enlarged figures in Figure 4). On the other hand, when q=-0.5e, HOMOs have smaller distribution on U1 due to the coulombic repulsion.

### 3.3. Switching in py QCA

**Figure 6:**
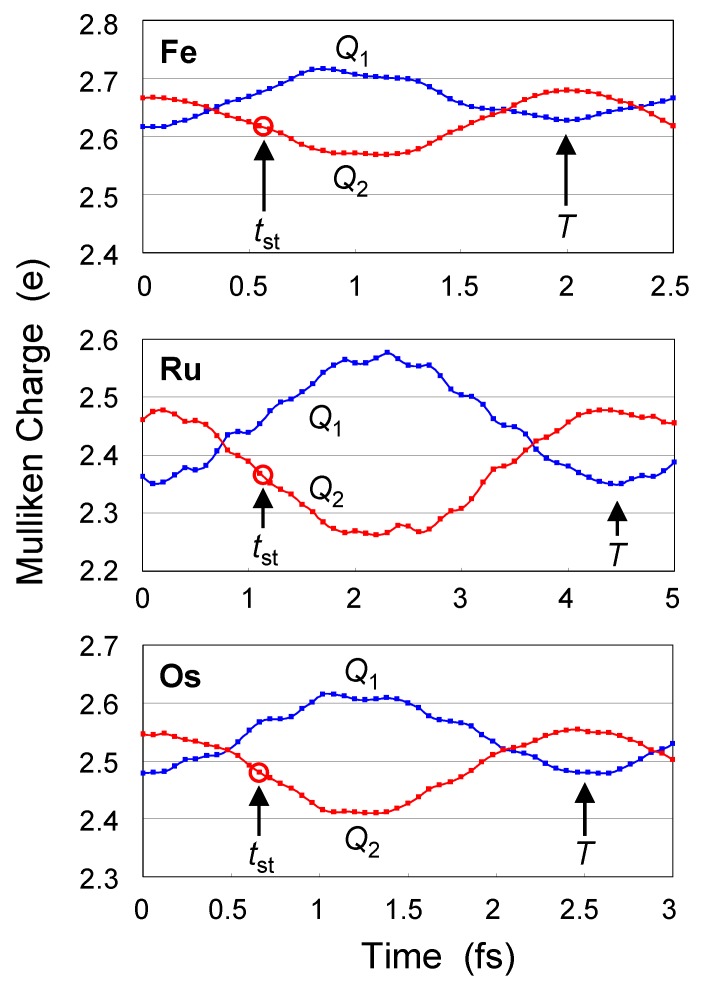
Dynamic behaviors of **py** complex upon the switch of input (*q* = +0.5e → *q* = -0.5e).

Figure 6 shows time evolution of $Q_{1}\left(t\right)$ and $Q_{2}\left(t\right)$ of **py** complexes after the switch of the input from *q* = +0.5e to *q* = -0.5e. The moment of the switch of input corresponds to *t* = 0. Summation of $Q_{1}$, $Q_{\mathrm{BL}}$, and $Q_{2}$ is always exactly +5, where $Q_{\mathrm{BL}}$ is the Mulliken charge of bridging ligand $Q_{\mathrm{BL}}$. Time evolution of $Q_{\mathrm{BL}}$ is not shown in this paper because BL has closed-shell electronic structure and time dependence of $Q_{\mathrm{BL}}$ is very small. As time flows after the switch, $Q_{2}$ decreases and $Q_{1}$ increases, namely, signal (electron) is transmitted from U1 to U2 by the coulombic repulsion.

Signal transmission time $t_{\mathrm{st}}$, which is the time when $Q_{1}\left(t_{\mathrm{st}}\right)=Q_{2}\left(0\right)$ and $Q_{2}\left(t_{\mathrm{st}}\right)=Q_{1}\left(0\right)$, is estimated as 0.6 fs (Fe) < 0.7 fs (Os) < 1.1 fs (Ru). After the signal transmission, periodic behavior is repeated with a period (*T*) of 2.0 fs (Fe) < 2.5 fs (Os) < 4.5 fs (Ru). From the Figures, values of signal amplitude *A* are estimated as 0.05 e (Fe) < 0.06 e (Os) < 0.10 e (Ru). All $t_{\mathrm{st}}$, *T*, and *A* are dependent on the kind of metal. From the viewpoint of operation speed of QCA device, Fe complex is most useful. On the other hand, from the viewpoint of signal power of QCA device, Ru complex is most useful.

Signal transmission time $t_{\mathrm{st}}$ is 1.1 fs at the maximum. On the other hand, the period *T* of nuclear motion is usually several hundreds fs. Therefore, nuclear vibration will have only a small influence on the signal transmission and can be neglected.

### 3.4. Switching in bpy QCA

**Figure 7:**
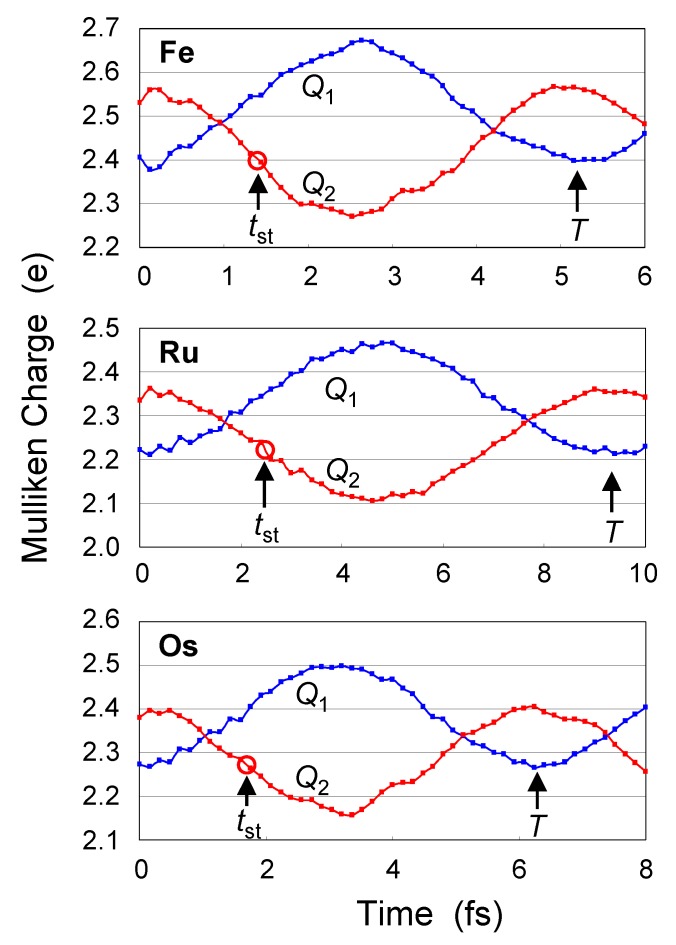
Dynamic behaviors of **bpy** complex upon the switch of input (*q* = +0.5e → *q* = -0.5e).

Figure 7 shows time-evolution of $Q_{1}\left(t\right)$ and $Q_{2}\left(t\right)$ of **bpy** complexes. Signal transmission time $t_{\mathrm{st}}$ is estimated as 1.4 fs (Fe) < 1.7 fs (Os) < 2.5 fs (Ru). After the signal transmission, periodic behavior is repeated with a period (*T*) of 5.2 fs (Fe) < 6.3 fs (Os) < 9.3 fs (Ru). These values of *T* are almost twice as large as those of **py** complexes, and are valid considering the difference in molecular size between **py** and **bpy** bridging ligands. The values of *A* are estimated as 0.11 e (Os) < 0.12 e (Ru) < 0.13 e (Fe). From the viewpoints of both operation speed and signal power of QCA device, Fe complex shows good result.

## 4. Discussion

### 4.1. Signal Period: T

Time-dependent part of Equation 6 is extracted as

(7) $$\begin{array}{c} \sum_{j,j^{\prime }\neq j}^{\mathrm{all}}-A_{ujj^{\prime }}\;\cos\left(2\pi t/T_{jj^{\prime }}\right), \end{array}$$

where

(8) $$\begin{array}{ccc} T_{jj^{\prime }} & = & 2\pi /\Delta \varepsilon_{jj^{\prime }}, \end{array}$$

(9) $$\begin{array}{ccc} A_{ujj^{\prime }} & = & \sum_{a\in u}^{\mathrm{Atom}}\sum_{\nu \in a}^{\mathrm{Basis}}\sum_{\mu }^{\mathrm{all}}\sum_{n}^{\mathrm{occ}.}d_{jn}d_{j^{\prime }n}\cdot c_{j\mu }c_{j^{\prime }\nu }\cdot s_{\mu \nu }. \end{array}$$

$T_{jj^{\prime }}$ and $A_{ujj^{\prime }}$ are the signal period and signal amplitude of unit *u* of the time evolution, respectively. The term $-A_{ujj^{\prime }}\cos\left(2\pi t/T_{jj^{\prime }}\right)$ represents the contribution of the interaction between $|\psi_{j}^{f}\rangle$ and $|\psi_{j^{\prime }}^{f}\rangle$ to the time evolution of $Q_{u}\left(t\right)$. In Table 2, two values of $T_{jj^{\prime }}$ are tabulated in order of $|A_{ujj^{\prime }}|$. For all complexes, (*j*, $j^{\prime }$) = (*H*, *L*) term is dominant so that the transmission behavior is almost determined by *H* and *L*, where *H* and *L* denote HOMO(*β*) and LUMO(*β*) when *q* = -0.5e. The values of the second largest $A_{ujj^{\prime }}$ are negligibly small. Thus, consideration of only (*H*, *L*) term is enough to reproduce Figure 6 and Figure 7. The $T_{jj^{\prime }}$ (or $\Delta \varepsilon_{jj^{\prime }}$) with the largest $A_{ujj^{\prime }}$ mainly determines the period (*T*) of the time evolution of Figure 6 and Figure 7. Orbital energies $\varepsilon_{j}^{f}$ are influenced by the strength of electric field originated from the input, but energy gaps $\Delta \varepsilon_{jj^{\prime }}$ between frontier MOs are almost determined by the interaction between metal atoms, bridging ligand, and ligands. Difference in the kind of metal atoms results in the difference in this interaction ($\Delta \varepsilon_{jj^{\prime }}$ and $T_{jj^{\prime }}$).

**Table 2:** Contribution of a set of (*j*, $j^{\prime }$) orbitals to the time-evolution of Mulliken charge. Two values of $T_{jj^{\prime }}$ (fs) are shown in order of $|A_{ujj^{\prime }}|$ (e). For all complexes, the set of (HOMO(*β*), LUMO(*β*)) gives the largest $A_{ujj^{\prime }}$.

		Unit 1				Unit 2		
		$j,j^{\prime }$	$A_{1jj^{\prime }}$	$T_{jj^{\prime }}$		$j,j^{\prime }$	$A_{2jj^{\prime }}$	$T_{jj^{\prime }}$
**py**	Fe	94β,95β	0.021	2.00		94β,95β	-0.026	2.00
		94β,96β	0.003	1.40		94β,96β	0.003	1.40
	Ru	112β,113β	0.052	4.47		112β,113β	-0.053	4.47
		112β,114β	0.001	1.47		109α,114α	0.002	0.94
	Os	84β,85β	0.031	2.48		84β,85β	-0.033	2.48
		84β,86β	0.002	1.32		84β,86β	0.002	1.32
**bpy**	Fe	114β,115β	0.065	5.15		114β,115β	-0.071	5.15
		114β,116β	0.004	1.93		114β,116β	0.005	1.93
	Ru	132β,133β	0.061	9.34		132β,133β	-0.061	9.34
		114α,135α	-0.001	0.44		131α,134α	0.001	0.93
	Os	104β,105β	0.056	6.26		104β,105β	-0.057	6.26
		104β,106β	0.002	1.62		103α,106α	0.003	1.12

### 4.2. Signal Amplitude: A

In dynamic behavior, signal amplitude (*A*) is almost determined by the value of $A_{uHL}$. $A_{uHL}$ is divided into two terms as

(10) $$\begin{array}{c} A_{uHL}=C_{uHL}D_{HL}, \end{array}$$

where

(9) $$\begin{array}{ccc} C_{uHL} & = & \sum_{a\in u}\sum_{\nu \in a}\sum_{\mu }c_{H\mu }c_{L\nu }s_{\mu \nu }, \end{array}$$

(10) $$\begin{array}{ccc} D_{HL} & = & \sum_{n}d_{Hn}d_{Ln}. \end{array}$$

Absolute values of $A_{uHL}$, $C_{uHL}$, and $D_{HL}$ are tabulated in Table 3. We can see that the order of $D_{HL}$ qualitatively corresponds to that of $A_{uHL}$. Therefore, the analysis of $D_{HL}$ is necessary for understanding the values of $A_{uHL}$. Although $D_{HL}$ is defined as a summation over all MOs *n* as seen in Equation 12, $d_{HH^{\prime }}d_{LH^{\prime }}$ term among all $d_{Hn}d_{Ln}$ terms has the dominant contribution to $D_{HL}$, where $H^{\prime }$ is HOMO(*β*) of initial stationary state (*q* = +0.5e), because $d_{Hn}$ is almost zero except for $n=H^{\prime }$. Additionally, although the values of $d_{HH^{\prime }}$ are almost an unit ($0.980<d_{HH^{\prime }}<0.999$) for all complexes, $d_{LH^{\prime }}$ is strongly dependent on the kind of metal. Consequently, we can qualitatively discuss the values of $|A_{uHL}|$ from that of $|d_{LH^{\prime }}|$. $H^{\prime }$ and *L* have been already shown in Figure 4 and Figure 5. In my previous paper, the values of $|A_{uHL}|$ were proportional to those of $|d_{LH^{\prime }}|$ since values of $C_{uHL}$ were almost constant for all systems [22]. In this paper, however, $|A_{uHL}|$ are not exactly proportional to those of $|d_{LH^{\prime }}|$ since the values of $C_{uHL}$ also depend on the kind of metal atoms.

**Table 3:** Absolute values of $A_{uHL}$, $C_{uHL}$, $D_{HL}$, $d_{HH^{\prime }}d_{LH^{\prime }}$, $d_{HH^{\prime }}$, and $d_{LH^{\prime }}$. Values of only U2 are shown because there is little difference between the values of U1 and U2, and dynamic behavior of U2 is more smooth and is suitable for analysis.

	py				bpy		
	Fe	Ru	Os		Fe	Ru	Os
$A_{uHL}$	0.026	0.053	0.033		0.071	0.061	0.057
$C_{uHL}$	0.295	0.429	0.352		0.372	0.445	0.401
$D_{HL}$	0.088	0.125	0.093		0.192	0.137	0.141
$d_{HH^{\prime }}d_{LH^{\prime }}$	0.088	0.125	0.093		0.192	0.137	0.141
$d_{HH^{\prime }}$	0.996	0.992	0.996		0.980	0.990	0.990
$d_{LH^{\prime }}$	0.088	0.126	0.094		0.195	0.139	0.143

In all complexes, larger distribution of $H^{\prime }$ is located on U1 (left-hand side). Similarly, larger distribution of *L* is on U1. For all complexes, $\psi_{L}^{f}\;\psi_{H^{\prime }}^{i}$ has larger distribution on U1 than on U2, so that the overlap integral $d_{LH^{\prime }}=\langle \psi_{L}^{f}|\psi_{H^{\prime }}^{i}\rangle$ has non-zero value in total.

About **py** complexes, we can see that $H^{\prime }$ and *L* of Ru complex have large distribution on the Ru metal but those of Fe complex have small distribution on the Fe metal from Figure 4. Therefore, the distribution of frontier orbitals of Ru complexes is strongly influenced by the switch of the input. Consequently, strongly deformed $H^{\prime }$ and *L* gives large $d_{LH^{\prime }}$ (and *A*). About **bpy** complexes, simple interpretation like **py** complexes are a little difficult because the difference in MO coefficients between metals of **bpy** complexes is smaller than that of **py** complexes. All complexes with **bpy** BL have small coefficients on BL and MOs distribute mainly on the metal atoms. Thus, signal amplitude *A* of **bpy** complexes is larger than that of **py** complexes and the difference in *A* between **bpy** complexes is small.

## 5. Conclusions

Dependence of the signal period *T*, the signal amplitude *A*, and the signal transmission time $t_{\mathrm{st}}$ on the kind of metal atoms was discussed taking [L${}_{5}$M-BL-ML${}_{5}$]${}^{5+}$ (M=Fe, Ru, Os, BL=pyrazine, 4,4’-bipyridine, L=NH${}_{3}$) as examples.

It was found that the order of $t_{\mathrm{st}}$ is Fe(0.6 fs) < Os(0.7 fs) < Ru(1.1 fs) and that of *A* is Fe(0.05 e) < Os(0.06 e) < Ru(0.10 e) for **py** complexes. For **bpy** complexes, $t_{\mathrm{st}}$ and *A* are Fe(1.4 fs) < Os(1.7 fs) < Ru(2.5 fs) and Os(0.11 e) < Ru(0.12 e) < Fe(0.13 e), respectively. **Bpy** complexes generally have stronger transmission signal but waste longer time than **py** complexes. Among all complexes, Fe complex with **bpy** BL shows the best results.

These results can be discussed from overlap integral $d_{LH^{\prime }}$ and energy gap $\Delta \varepsilon_{HL}$ of molecular orbitals. Complexes with large $\Delta \varepsilon_{HL}$ have small *T*. The values of $\Delta \varepsilon_{HL}$ can be explained from the orbital interaction between M, BL, and L. On the other hand, *A* can be explained from the asymmetry of frontier orbitals. MOs with large coefficients on M atom tend to be strongly affected by the switch of the input. Therefore, overlap integral $d_{LH^{\prime }}$ tends to be large.
